# Complexity and developmental changes in the expression pattern of claudins at the blood–CSF barrier

**DOI:** 10.1007/s00418-012-1001-9

**Published:** 2012-08-11

**Authors:** Ingrid Kratzer, Alexandre Vasiljevic, Catherine Rey, Michelle Fevre-Montange, Norman Saunders, Nathalie Strazielle, Jean-François Ghersi-Egea

**Affiliations:** 1 Inserm U1028, CNRS UMR 5292, Lyon Neuroscience Research Center, Lyon-1 University, 69008 Lyon, France; 2 East Pathological Center, Lyon Public Hospitals, Lyon, France; 3 ProfilXpert, UNIV-US7 INSERM-UMS 3453 CNRS Lyon, Lyon, France; 4 Department of Pharmacology, University of Melbourne, Melbourne, Australia; 5 Brain-i, Lyon, France; 6 Neurooncology and Neuroinflammation Team, INSERM U1028, CNRS UMR 5292, Lyon Neuroscience Research Center, Faculté de Médecine Laennec, 7 rue G. Paradin, 69372 Lyon Cedex 08, France

**Keywords:** Blood–brain barrier, Brain development, Choroid plexus, Tight junction

## Abstract

The choroid plexus epithelium controls the movement of solutes between the blood and the cerebrospinal fluid. It has been considered as a functionally more immature interface during brain development than in adult. The anatomical basis of this barrier is the interepithelial choroidal junction whose tightness has been attributed to the presence of claudins. We used quantitative real-time polymerase chain reaction, Western blot and immunohistochemistry to identify different claudins in the choroid plexuses of developing and adult rats. Claudin-1, -2, and -3 were highly and selectively expressed in the choroid plexus as compared to brain or parenchyma microvessels and were localized at epithelial junctions. Claudin-6, -9, -19, and -22 also displayed a previously undescribed choroidal selectivity, while claudin-4, -5, and -16 were enriched in the cerebral microvessels. The choroidal pattern of tight junction protein expression in prenatal brains was already complex and included occludin and zonula occludens proteins. It differed from the adult pattern in that the pore-forming claudin-2, claudin-9, and claudin-22 increased during development, while claudin-3 and claudin-6 decreased. Claudin-2 and claudin-11 presented a mirror image of abundance between lateral ventricle and fourth ventricle choroid plexuses. Imunohistochemical analysis of human fetal and postnatal brains for claudin-1, -2, and -3 demonstrated their early presence and localization at the apico-lateral border of the choroid plexus epithelial cells. Overall, choroidal epithelial tight junctions are already complex in developing brain. The observed differences in claudin expression between developing and adult choroid plexuses may indicate developmental differences in selective blood–cerebrospinal fluid transport functions.

**Electronic supplementary material:**

The online version of this article (doi:10.1007/s00418-012-1001-9) contains supplementary material, which is available to authorized users.

## Introduction

A tight regulation of the neural cell microenvironment is mandatory for efficient neuronal activities. Cerebral homeostasis largely results from the ability of both the blood–brain barrier (BBB) at the brain microvascular endothelium and the blood–cerebrospinal fluid barrier (BCSFB) at the epithelium of the choroid plexuses (CPs), to control the composition of the CSF and cerebral extracellular fluid. Tight junction (TJ) proteins that link the cells forming these blood–brain interfaces form the anatomical basis for this control by preventing non-specific paracellular leakage between blood and the cerebral fluids. Influx and efflux transporters located at both barriers allow nutrient supply to the brain, while excluding a wide range of potentially deleterious compounds from this organ. The CPs, which are located in the different ventricles of the brain fulfill additional specific functions. They are a source of trophic factors during brain development, are responsible for controlled secretion of CSF, and are major detoxifying organs within the brain (Davson and Segal 1996; Saunders et al. 2008; Strazielle and Ghersi-Egea 2000; Zappaterra and Lehtinen 2012).

The brain is especially vulnerable during development, and perinatal brain injury can lead to altered myelination, reduced neurogenesis or inappropriate neuronal network organization with dramatic consequences throughout life (Dammann and Leviton 1999; Stolp et al. 2011). The blood–brain interfaces are often considered immature in the developing brain due to a high protein concentration in the CSF (e.g. Adinolfi 1985). However, electron microscopy and tracer experiments in rodents and marsupials indicate that the paracellular pathway is already restricted in early developing brain (Ek et al. 2006; Johansson et al. 2006), and alternative explanations based on CSF space volume, CSF turnover, and transcellular transport of specific proteins have been brought forward to explain the presence of proteins in the CSF of developing animals (Johansson et al. 2008b). Transfer of exogenous polar compounds and some endogenous proteins between the blood and the CSF has been demonstrated in the developing rat brain, which partly occurs by diffusion (Habgood et al. 1992). A transcellular route has been described for both proteins and small lipid insoluble molecules during development (Balslev et al. 1997; Liddelow et al. 2009), but further studies are required to resolve whether there is also a paracellular component of this transfer before a definitive conclusion can be reached.

Unraveling the complexity of TJs is important to appreciate the level of neuroprotection provided by the blood–brain interfaces at different stages of brain maturation, especially during the perinatal period, and to understand cerebral drug bioavailability in the context of pediatric treatments. The tightness of the cellular junctions results from the complex interplay of transmembrane and accessory proteins. The transmembrane proteins of the claudin (Cld) family play a crucial role in determining the efficiency and selectivity of TJs. In mammals Cld-5 is the main Cld identified in BBB tight junctions, which also comprise Cld-3 and -12 (Morita et al. 1999b; Nitta et al. 2003; Wolburg et al. 2003). The molecular identity of the TJ-forming Clds at the BCSFB remains more elusive. So far Cld-1/3,-2, and -11 have been described in the rodent or murine CP epithelium (Lippoldt et al. 2000; Wolburg et al. 2001). In human, Cld-5 was also shown to localize in intercellular junctions of the cerebral microvascular endothelium (Virgintino et al. 2004), but no data exist concerning the composition of TJ that link the choroidal epithelial cells forming the BCSFB.

To decipher the molecular composition and understand the complexity of TJs at the BCSFB in the developing rat brain, we analyzed the pre- and post-natal developmental profile of Cld expression in the CP, in comparison to the BBB-forming microvessels (MV), and determined the cellular localization of these proteins. On the basis of the current knowledge of TJ proteins in the brain, and preliminary transcriptomic analyses we performed on choroidal tissue, we selected 14 members of the Cld family. Occludin and the three zonula occludens (ZO) peripheral membrane proteins were also included. We report the presence of hitherto undescribed Cld members associated with junctional complexes of the BCSFB and BBB, and show differences in the developmental patterns of TJ proteins in the CP of the lateral and fourth ventricle. We finally demonstrate that knowledge of the early choroidal expression of Clds can be extended to the human BCSFB.

## Materials and methods

### Tissue collection

Animal care and procedures were conducted according to the guidelines approved by the French ethical committee (decree 87-848) and by the European Community (directive 86-609-EEC). Sprague–Dawley rats, either adult males (250 g), pregnant time-dated females or females with litters were obtained from Janvier (Le Genest Saint Isle, France). All animals were kept under similar conditions in standard cages, with free access to food and tap water under a controlled environment (12 h day/light cycle). Animals were euthanized under isofluorane anesthesia by decapitation and their brains were rapidly excised and placed at 4 °C in Krebs–Ringer buffer (in mM: 135 NaCl, 4 KCl, 2.2 CaCl_2_, 1.2MgCl_2_, 6 NaHCO_3_,10 HEPES, 5 glucose, pH 7.4). The two lateral ventricle choroid plexuses (LCVP) and the fourth ventricle choroid plexus (4VCP) were dissected individually from postnatal day 2 (P2), postnatal day 9 (P9) and adult rats at 4 °C under stereomicroscope as previously described and illustrated (Strazielle and Ghersi-Egea 1999, 2000). Nineteen day-old embryos (E19) were removed one by one from the mother, which was kept under isoflurane anesthesia on a heated pad and were used for brain sampling and further microdissection of the CPs as described above. CPs collected from several animals (between 2 and 5, depending on the developmental stage) were pooled. Following CP isolation, brain cortices were sampled at all stages and cleaned of apparent meninges under a stereomicroscope. These tissues were either used as homogenates or for RNA extraction at all stages of development, or for MVs preparation at P9 and adult stages. The cortical MVs were isolated and assessed for purity as previously described (Gazzin et al. 2008). Briefly, cortices were homogenized in a Dounce-type glass–glass homogenizer after the addition of 5 vol/g tissue of 1 % bovine serum albumin (BSA)-supplemented Krebs–Ringer. The MVs were separated from larger vessels and brain parenchyma material by a sequence of a 17.5 % 70 kDa-Dextran gradient followed by filtering steps through decreasing pore diameter mesh sieves. The microvessel fraction retained on the 40-μm sieve was recovered in 0.1 % albumin in Krebs–Ringer buffer. All steps were performed at 4 °C. For gene expression analysis, the tissue collection and MV isolation were performed under RNase-free conditions. Samples collected for gene expression analyses and Western blot (WB) were snap-frozen in liquid nitrogen and kept at −80 °C. For immunohistochemistry, brains sampled from E19 to adult animals were snap-frozen in isopentane at −50 °C, embedded in Tissue-Tek (Sakura Finetek Europe, The Netherlands) and stored at −80 °C.

Human fetal brain tissues were obtained from autopsies performed at the Centre de Pathologie Est, Groupement Hospitalier Est, Hospices Civils de Lyon, France, after obtaining an informed consent from the parents.

### Quantitative RT-PCR

Total RNA was isolated from four batches of 4VCP, LVCP as well as cortices sampled from E19, P2, P9 and adult rats using the RNeasy^®^ Micro Kit (Qiagen, Valencia, CA, USA), and DNAse-treated according to the manufacturer’s protocol. For comparative purpose, RNA was also isolated from two batches of MVs prepared from P9 and adult rat brain. Total RNA was quantified using OD_260nm_ on a NanoDrop 2000c spectrophotometer (ThermoScientific, Baltimore, MA, USA) and quality was assessed with the Agilent 2100 Bioanalyser (Agilent Technologies, Palo Alto, CA, USA). RNA (1 μg) was spiked with 25 pg of bacterial AraB RNA from *E. coli* used as an external standard (GE Healthcare Bio-Sciences Freiburg, Germany) and reverse transcribed using the iScript Reverse Transcription Supermix for RT-qPCR (Bio-Rad, Hercules, CA, USA). This external bacterial standard was used for normalization as the expression of conventionally used house-keeping genes including glyceraldehyde-3-phosphate deshydrogenase or hypoxanthine-phosphoribosyl transferase proved to be variable between tissues or developmental stages. Quantitative real-time PCR (qRT-PCR) was performed with the LightCycler FastStart-DNA Master SYBR Green I kit and the LightCycler^®^ 1.5 Instrument (Roche Diagnostics GmbH, Mannheim, Germany). All primers were designed using NCBI Primer-BLAST and selected to generate amplicons with a length of 100–200 bp (Online Resource 1). The LightCycler experimental run protocol consisted of an initial *Taq* activation at 95 °C, for 8 min followed by a “touch down” amplification program. The first cycle of the program consisted of 15 s at 95 °C, 5 s at 68 °C and 8 s at 72 °C. The annealing temperature was reduced by 0.5 °C every cycle until 62 °C was reached. This was followed by generic PCR amplification for 27 additional cycles keeping the annealing temperature at 62 °C. Melting-curve analysis was then performed to verify the amplification of a single product with a specific melting temperature. MgCl_2_ concentration was optimized for each gene and negative PCR controls without cDNA template were included in every run. A standard curve was generated using the LightCycler^®^ Software 4.1 by non-linear regression analysis of crossing points (Cp) measured from serial dilutions of a cDNA pool for each gene analyzed and for the external standard AraB. Cp values of unknown samples were used with the appropriate standard curve to determine in each sample the relative cDNA concentration of the target gene. Potential variability in sample-to-sample reverse transcription efficiency and RT-PCR processing was corrected by normalizing the data to AraB expression.

To provide an approximate ranking of the different Cld gene product abundance, expression levels of all genes were estimated first in a reference sample, arbitrarily chosen as P2 LVCP #3 as follows: AraB efficiency^AraB CP^/Target efficiency^Target CP^, where efficiencies of amplification were calculated from the linear part of the standard curves using the LightCycler^®^ Software 4.1 (http://www.roche-applied-science.com/sis/rtpcr/lightcycler/index.jsp?id=lct_0106010302). The obtained values were expressed as a percentage of Cld-1 value after correction for variation in the size of amplification product. For each target gene, the expression levels of all individual samples were finally expressed relative to the value of the reference sample.

The significance of differences in expression levels between tissues or between developmental stages was assessed for each gene by one-way ANOVA followed by a Tukey’s multiple comparison test. The significance of difference in expression levels between age-matched 4VCP and LVCP was assessed by two-tailed paired Student’s *t* test.

### Primary antibodies

Polyclonal rabbit anti-Cld-1 Ab (pAb) (51-9000, not cross-reacting with Cld-3), rabbit anti-Cld-2 pAb (51-6100), rabbit anti-Cld-3 pAb (34-1700), rabbit anti-Cld-4 pAb (36-4800), rabbit anti-Cld-5 pAb (34-1600) and the mouse anti-Cld-5 monoclonal antibody (mAb) (35-2500) were purchased from Zymed Laboratories (Invitrogen, Carlsbad, CA). Rabbit anti-Cld-9 pAb (16196-1-AP) was bought from Protein Tech Group (Chicago, IL). Rabbit anti-Cld-11 pAb (AP06062PU-N) was ordered from Acris Antibodies (SanDiego, CA). Antibody quality was assessed by Western blot of CPs or MVs. Only one specific band for Cld-1, -2, -3 (all 22 kDa) or Cld-9 (25 kDa) was observed in adult LVCP. The anti-Cld-4 pAb and anti-Cld-5 monoclonal Ab detected only one band (22 kDa) in adult MVs (Online Resource 2). The rabbit anti-Cld-5 antibody also selectively labeled parenchyma MVs and was used for double immunostaining with the endothelial cell antibody RECA-1. The rabbit anti-Cld-19 pAb, custom-produced by Zymed Laboratories as an IgG preparation, was a kind gift of Dr. Yu (Angelow et al. 2007). In our hands, the antibody stained the tight junctions of the thick ascending limb of Henle on rat kidney sections, as previously described in mouse kidney cortex (Angelow et al. 2007). It was not applicable to Western blot analysis of cerebral preparations. The mouse anti-RECA-1 mAb (MCA970GA), directed against a rat endothelial cell-surface antigen, and the rabbit anti-actin pAb (A-2066) were purchased from AbD Serotec (Oxford, UK), and Sigma (St-Louis, MO, USA), respectively.

### Immunohistochemical analysis of claudins in rat brain

Rat brain sections (10 μm thick) were cut in a cryostat, mounted on glass-slides, air-dried at room temperature (RT), and immediately used for IHC or kept at −20 °C. Slices were fixed in acetone/methanol (V/V) at −20 °C for 90 s except for Cld-4. For this protein, sections were fixed in 4 % paraformaldehyde at RT for 10 min and microwave-treated (3 × 5 min at 600 W) in 0.01 M citrate buffer, pH 6 for epitope retrieval. After 1 h of saturation at RT in a blocking solution containing 0.2 % BSA, 0.2 % Triton X-100 and 10 % normal goat serum (NGS) in phosphate buffered saline (PBS, pH 7.4), sections were incubated at 4 °C overnight with primary antibodies diluted in a PBS solution containing 1 % BSA, 0.3 % Triton, 1 % NGS. Primary antibodies were diluted and used at a final concentration of 0.625 μg/ml for Cld-1, -2, -3,- 4. The anti-Cld-5 mAb and pAb were used at 1.25 μg/ml and 0.625 μg/ml, respectively. Cld-9 pAb was diluted to 0.383 μg/ml and Cld-19 pAb to 4 μg/ml. In some cases, double labeling was performed with one of the anti-Cld pAb and the anti-RECA-1 mAb used at a final concentration of 2.5 μg/ml. After 5 washes for 10 min at RT in the Ab-solution without NGS, sections were incubated with either Alexa Fluor^®^ 488-conjugated secondary goat-anti-mouse Ab (A-10667), Alexa 555^®^-conjugated goat-anti-mouse secondary Ab (A-21424) or Alexa 555^®^-conjugated secondary goat-anti-rabbit Ab (A-21428) all from Invitrogen at a final concentration of 2 μg/ml at RT for 1 h. Diamidine-2-phenylindole-dihydrochloride (DAPI, 236276 from Roche Diagnostics) was used as fluorescent nuclear stain at 0.1 μg/ml in PBS. Sections were washed again four times in Ab-solution without NGS and rinsed once in PBS. Negative controls were performed by omitting the primary Ab. After mounting the slides with Fluoroprep (bioMerieux, Marcy l’Etoile, F), immunofluorescence was viewed and analyzed using a Zeiss fluorescence microscope equipped with a Digital Camera and the software AxioVs40 V 4.7.2.0. For direct comparison of expression and localization between either developmental stages or between 4VCP and LVCP at a given stage, appropriate sections were mounted on the same slide and treated simultaneously under identical conditions. Pictures were taken at the same exposure time to assess potential expression differences. The staining specificity of the custom-produced Cld-19 pAb was evaluated using a blocking peptide on brain slices. The IgG preparation was incubated with a tenfold molar excess of peptide that corresponds to the epitope recognized by the Ab (H-ERANSIPQPYRSGPSTAAREYV-OH) at 4 °C for 8 h under agitation. The staining protocol was then performed as described above.

### Immunohistochemical analysis of claudins in human fetal brain

Fetal brains were fixed in 4 % formaldehyde. The duration of fixation is provided in Table 1. Coronal slices of the supratentorial brain and horizontal slices of the brainstem and cerebellum were performed and embedded in paraffin. Sections (4 μm thick) were cut from the blocks with a Leica microtome (Leica Instruments GmbH, Hubloch, Germany) and transferred to SuperFrost Plus slides (VWR International bvba, Leuven, Belgium) for morphological and immunohistochemical studies. Hemalum phloxin saffron (HPS) staining was performed for morphological analysis. An automated IHC analysis of the sections was performed on a BenchMark^®^ XT (Ventana Medical Systems Inc, Tucson, AZ, USA) using anti-Cld antibodies at a final concentration of 2.5 μg/ml according to the manufacturer’s protocol, and revealed by avidin–biotin–peroxidase complex, and a Ventana kit including DAB reagent and an amplification system. Sections were counterstained with hematoxylin. The localization, continuity and intensity of Cld expression were assessed in all 13 cases.

**Table 1:** Description of human samples included in the study and claudin immunopositivity evaluation

Case	WD	Clinical history	Fixation (days)	CLD-1		CLD-2		CLD-3	
				LVCP	4 VCP	LVCP	4 VCP	LVCP	4VCP
1	8	Spontaneous abortion	3	+ CA	+ CA	+ DA	+ DA	+ CA	+ CA
2	10	Limb malformations	3	+ CA	+ CA	+ DA	+ DA	+ CA	+ CA
3	10	Spontaneous abortion	7	+ CA	+ CA	+ DA	+ DA	+ CA	+ CA
4	16	Spontaneous rupture of membranes	13	+ CA	+ CA	+ CA	+ CA	+ CA	+ CA
5	18	Twin pregnancy Spontaneous rupture of membranes	43	+ CA	+ CA	+ CA	+ CA	+ CA	+ CA
6	20	Acute chorioamniotitis	40	+ CA	+ CA	+ CA	+ CA	± CA	± CA
7	20–21	Twin pregnancy Late spontaneous abortion	35	+ CA	+ CA	+ CA	+ CA	+ CA	+ CA
8	21	Acute chorioamniotitis	67	+ CA	+ CA	+ CA	+ CA	± CA	± CA
9	22	Acute chorioamniotitis	8	+ CA	+ CA	+ CA	+ CA	+ CA	+ CA
10	28–29	Premature, pulmonary hypoplasia, refractory hypoxia	58	+ CA	+ CA	+ CA	+ CA	0	0
11	38	Postpartum death, SIDS	54	+ CA	+ CA	+ CA	+ CA	+ CA	± DA
12	39	Postpartum death, SIDS	17	+ CA	+ CA	+ CA	+ CA	+ CA	+ CA
13	2 months and 17 days post-natal	SIDS	38	+ CA	+ CA	+ CA	+ CA	+ CA	+ CA

Immunolabeling was scored as follows: 0: no staining, ± : weak staining, + and +: definite staining. *C* continuous, *D* discontinuous, *A* apico-lateral

*LVCP* choroid plexus from the lateral ventricle, *4VCP* choroid plexus from the fourth ventricle, *WD* weeks of development, *SIDS* sudden infant death syndrome

### Protein content

Choroidal tissues and brain cortices were homogenized in Cell lysis buffer (9803 from Cell Signaling, Boston, MA) containing 1 mM phenylmethylsulfonyl fluoride (PMSF, igma, St. Louis, MO) with 10–15 strokes in a glass–glass homogenizer. Protein concentrations were determined by the method of Peterson (1977) using BSA for the standard curve.

### Western blotting

Proteins were separated on 10 % Bis–Tris Criterion XT gels (Bio-Rad, Hercules, CA, USA) run in MOPS buffer (Bio-Rad) at 80 V for 10 min, followed by 120 V for 2 h. The gel was blotted to 0.45 μm Whatman PROTRAN nitrocellulose membrane (# NBA085C, Perkin Elmer, Waltham, MA) in Tris–glycine transfer buffer (Euromedex, Souffleweyersheim, F), containing 20 % methanol for 50 min. After assessing transfer efficiency by Ponceau-red staining, the membrane was cut longitudinally to separate actin from the lower molecular weight Clds and incubated in a saturation buffer (5 % skimmed milk, 0.1 % Tween 20 in Tris-buffered saline; TBS) at RT for 1 h. When antibody quality was tested, the membranes were kept in one piece. All primary anti-Cld antibodies were diluted in saturation buffer at 1 μg/ml except Cld-9, which was used at 0.307 μg/ml. The rabbit anti-actin pAb was used at a final dilution of either 0.533 or 0.267 μg/ml, depending on the amount of protein loaded. Membranes were incubated at 4 °C overnight, then washed 3 × 10 min in TBS containing 0.1 % Tween-20 (TBST) at RT. Horseradish peroxidase-conjugated secondary antibodies from Jackson ImmunoResearch (Baltimore, MA, USA) were either goat-anti-rabbit (111-035-045) used at a final concentration of 0.04 μg/ml or goat-anti mouse (115-036-003) used at 0.08 μg/ml. After two washes in TBST and a final one in TBS, chemiluminescent HRP substrates (Immobilon Western from Millipore, Molsheim, France, for Clds, and Pierce ECL substrate from Perbio Science, Brebière, France, for actin) were used and membranes were exposed to X-ray films (Biomax, Kodak Rochester, NY).

## Results

**Fig. 1:**
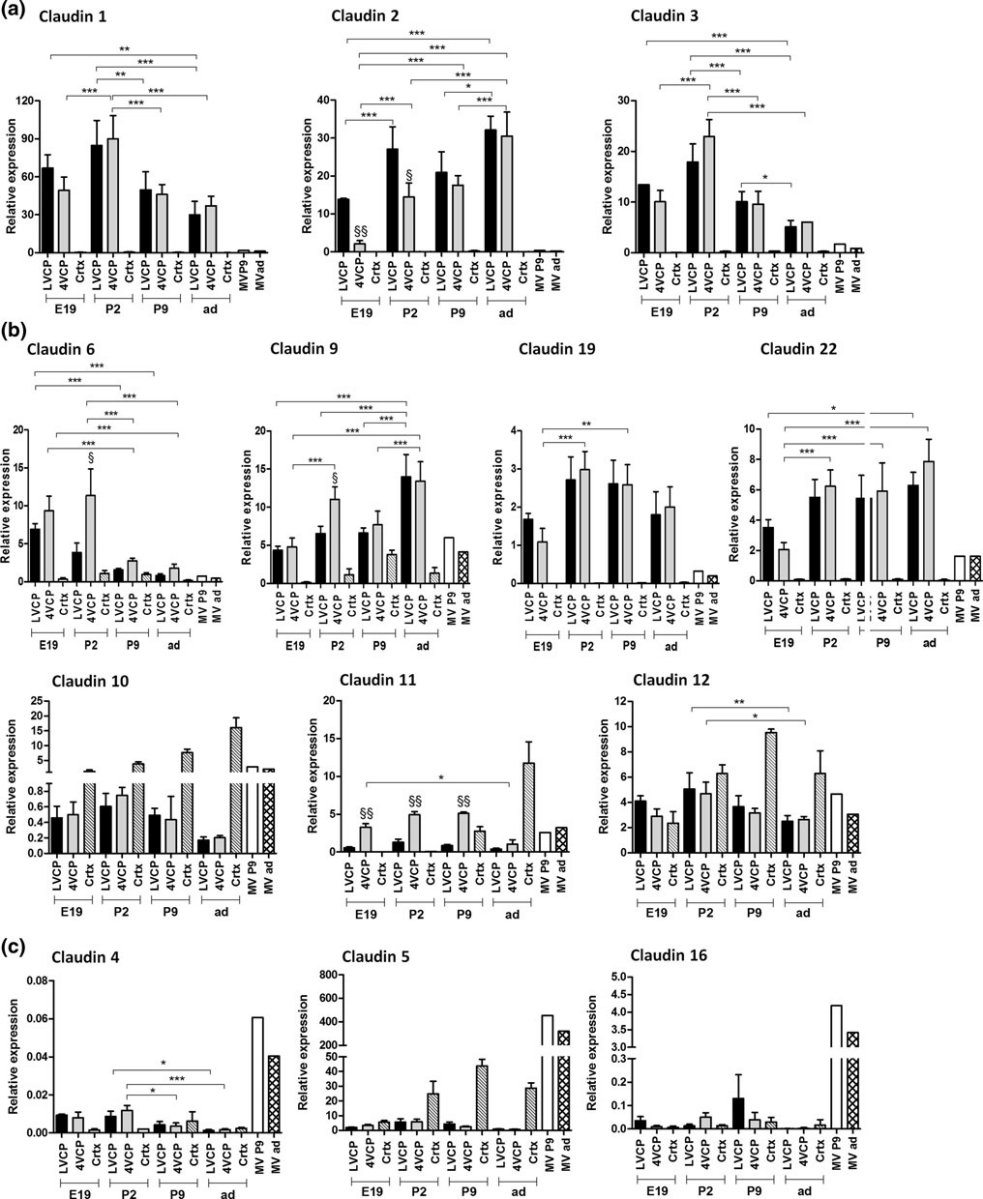
Developmental analysis of claudin transcript levels in choroid plexuses, cerebral cortex and cerebral microvessels of the rat. RT-PCR was performed on tissues sampled at four developmental stages. The results, expressed relative to the bacterial AraB gene added as an external standard, are shown as mean ± SD, *n* = 4, except for MV, for which values represent the mean of two preparations. Gene expression levels did not differ by more than 13.8 % between the two age-matched MV preparations. The results are expressed as arbitrary units, and are all normalized to the expression level found for Cld-1 in one sample of LVCP obtained from P2 animals, and set arbitrarily at 100 (see “Materials and methods” for details of calculations). **a** Clds displaying the highest specific expression in CP throughout development. **b** Clds expressed at lower, but significant levels in the CP by comparison to other brain preparations. Some displayed a strong specificity for the choroidal tissue. **c** Clds specifically enriched in the MV preparations, see “Results” for a detailed description of the data. *, **, ***Statistical differences in expression levels between developmental stages, analyzed by one-way ANOVA followed by a Tukey’s multiple comparison test, *p* < 0.05, 0.01, 0.001, respectively. For clarity, only differences observed for LVCPs and 4VCPs are shown on the graphs. ^§^, ^§§^Statistical differences in expression levels between age-matched 4VCPs and LVCPs, two-tailed paired Student’s *t* test, *p* < 0.05 and 0.01, respectively. Other relevant statistical analyses are stated in “Results”. *LVCP* and *4VCP* choroid plexus of the lateral and fourth ventricle, respectively, *Crtx* cerebral cortex, *MV* cerebral microvessels, *E19* 19-day-old embryo, *P2* and *P9* 2-day-old and 9-day-old, respectively; *ad* adult

The expression of TJ-associated proteins was assessed in rats at E19, P2, P9 and adult stages in LVCPs and 4VCPs by qRT-PCR. For comparison, the expression levels of these genes were also measured in cortical tissues at all stages, and in cortical MVs isolated from P9 and adult brain (Figs. 1, 7). Differences in *y* scale values between graphs provide an approximation of the respective abundance of the various gene products (see “Materials and methods”). Overall, Cld-1, -2 and -3 were the most highly expressed Clds in the choroidal tissue and displayed a distinct specificity for this tissue in comparison to age-matched MVs and cortices (Fig. 1a). The latter tissue displayed 10- to 100-fold lower expression levels than CPs (*p* < 0.001). Cld-6, -9, -10, -11, -12, -19 and -22 were expressed at lower, but still significant levels in the choroidal tissue (Fig. 1b). While Cld-19 and Cld-22 displayed a high degree of choroidal specificity at all stages investigated (statistically different from age-matched cortex mRNA levels, *p* < 0.001 for P2, P9, adult), and Cld-9 followed a similar pattern of choroidal enrichment (*p* < 0.05 for LVCP and 0.01 for 4VCP at E19, *p* < 0.001 at P2 and in adult), Cld-6 was found enriched in CP as compared to cortex preparations only in the perinatal developmental stages (*p* < 0.001 at E19 and 0.001 for 4VCP at P2, Fig. 1b). Cld-4, -5, and -16, whose expression in the choroidal tissue was very low, were rather selectively enriched in the MVs forming the BBB (Fig. 1c). Cld-14 was not detected in any sample of CP, cortex and MV. Its mRNA was found in liver, used as positive control (data not shown). The developmental qRT-PCR analysis of Clds in CPs revealed overall high expression levels in the tissues sampled at prenatal and postnatal stages. We observed however variations in the choroidal expression and localization of Clds during development and investigated them in more detail using protein analysis when antibodies of sufficient quality were available.

### Claudin-1 and -3 expression is high in both types of choroid plexus in the developing brain

**Fig. 2:**
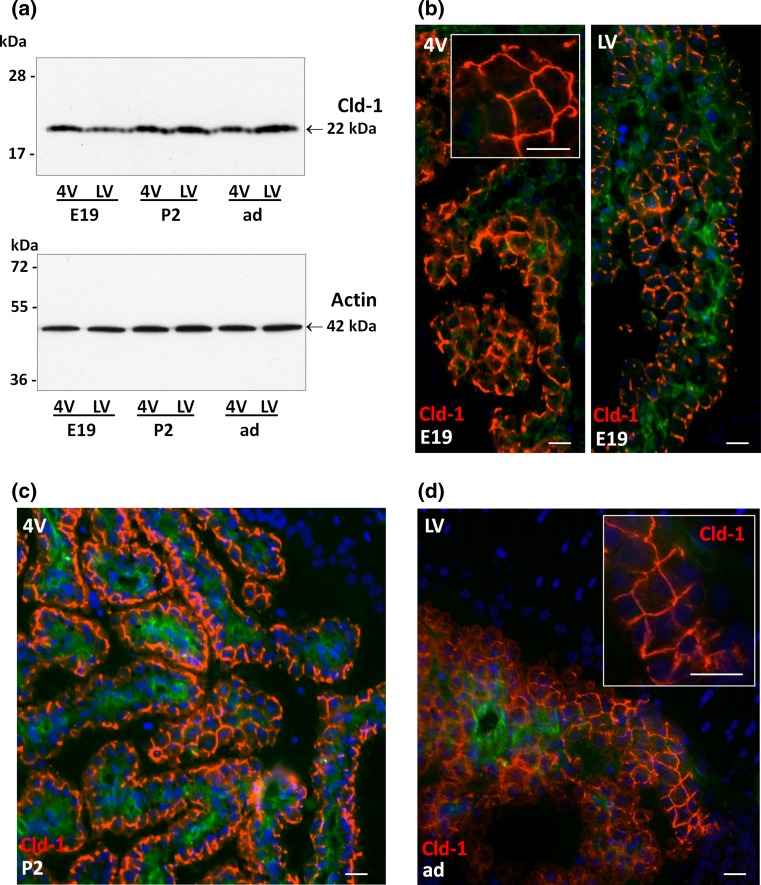
Western blot analysis and cellular distribution of claudin-1 in choroid plexus during rat brain development. **a** Representative Western blot of LVCP and 4VCP dissected from E19, P2, and adult animals (2 μg per lane). Cld-1 protein expression was steady throughout development in both types of plexuses (*upper panel* band at 22 kDa). Actin used as a loading indicator is shown in the *lower panel* (42 kDa band). **b**–**d** Cld-1 immunoreactivity (*red*) in CPs of the developing and adult brain. A strong signal was associated with epithelial cell membranes of both LVCP and 4VCP in E19 animals (**b** *left* and *right panel*, respectively). A similar signal was observed at later stages, as illustrated for 4VCP in a P2 animal (**c**), or LVCP in an adult rat (**d**). The *inserts* in **b** and **d** highlight the typical honeycomb pattern of choroidal epithelial cell junctions. Double immunolabeling with the anti-RECA-1 Ab (*green*) allowed visualizing choroidal vessels, and DAPI was used for nuclei staining. *Scale bars* 20 μm. *LV* and *4V* choroid plexus from the lateral and fourth ventricle, respectively, *E19* 19-day-old embryo, *P2* 2-day-old, *ad* adult

Quantitative RT-PCR analysis showed that Cld-1 and -3 are already highly expressed in both LVCPs and 4VCPs of E19 and P2 rats compared to P9 and adult animals. Cld-1 and Cld-3 mRNA levels were not statistically different between 4V and LVCP at all stages investigated (Fig. 1a). Western blot analysis (see method section and Online Resource 2 for details on the quality of the antibodies used) confirmed the expression of Cld-1 in CPs of E19 and P2, and showed no obvious difference in protein levels between 4VCP and LVCP, or between stages (Fig. 2a). This result and all subsequent Western blot analyses presented have been confirmed on at least three batches of tissues. The cellular protein localization was then determined by IHC. Cld-1 was found strictly associated with the interepithelial junctions of both 4VCP and LVCP at all stages investigated (Fig. 2b–d). Cld-1 was not detected in the choroidal or parenchymal vessels at any stage of development.

**Fig. 3:**
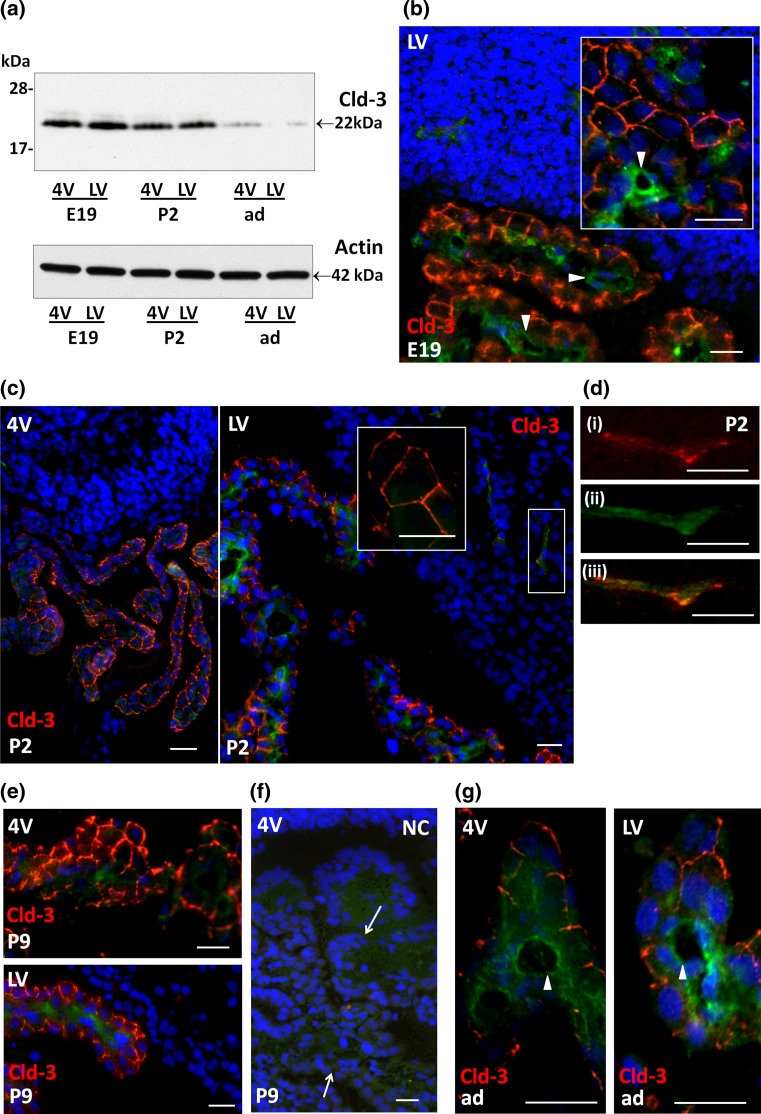
Western blot analysis and cellular distribution of claudin-3 in choroid plexus and cerebral parenchyma during rat brain development. **a** Representative Western blot of LVCP and 4VCP dissected from E19, P2, and adult animals (10 μg per lane). Cld-3 protein levels decreased in both types of CP during development (*upper panel* band at 22 kDa). Actin used as a loading indicator is shown in the lower panel (42 kDa band). **b**–**g** Cld-3 immunoreactivity (*red*) in the developing and adult brain. Double immunolabeling with anti-RECA-1 Ab (*green*) allowed visualizing choroidal and parenchymal vessels (e.g. arrowheads in **b**, **g**), and DAPI was used for nuclei staining. During perinatal stages of development, immunodetection revealed a distinct signal associated with the CP epithelial cell membranes as illustrated for LVCP in E19 (**b**), or in P2 animals (**c**). The *inserts* in **b** and **c** highlight the typical honeycomb pattern of choroidal epithelial cell junctions. The area delimited by the *rectangle* in **c**, is enlarged in **d**, which shows Cld-3 in a parenchymal vessel of the developing brain (*i* Cld-3 (*red*), *ii* RECA-1 (*green*) and *iii* merge staining). Cld-3 immunoreactivity was observed at the epithelial junctions in CPs of P9 animals (**e**), and could still be detected in adult CP (**g**). **f** A negative control (NC), run in the absence of primary antibody, for a P9 animal (*arrow* CP). *Scale bars* 20 μm. Abbreviations as in Fig. 2

Western blot analysis of Cld-3 in CPs also confirmed the expression of the protein in E19 and P2 animals, and additionally showed a clear decrease of Cld-3 protein level in the CPs of adult animals (Fig. 3a). This reflects the strong and statistically significant down-regulation of Cld-3 mRNA observed between P2 and adult stages (Fig. 1a). IHC analysis showed that the protein is associated with the inter-epithelial junctions of both 4VCP and LVCP in developing animals (Fig. 3b, c, e, f). Cld-3 immunoreactivity was also observed in both CPs in the adult (Fig. 3g), with a lower intensity than in developing animals. The protein was not detected in the choroidal vessels (e.g. arrowheads in b). In contrast, parenchymal vessels were labeled (Fig. 3d), in line with the previously reported Cld-3 immunoreactivity in parenchymal vessels of the adult mouse brain (Wolburg et al. 2003). Cld-3 labeling was however weaker in parenchymal vessels than in the CP epithelium at all stages investigated.

### The developmental profile of claudin-2 and -11 expression differs between the choroid plexuses of the lateral and fourth ventricles

**Fig. 4:**
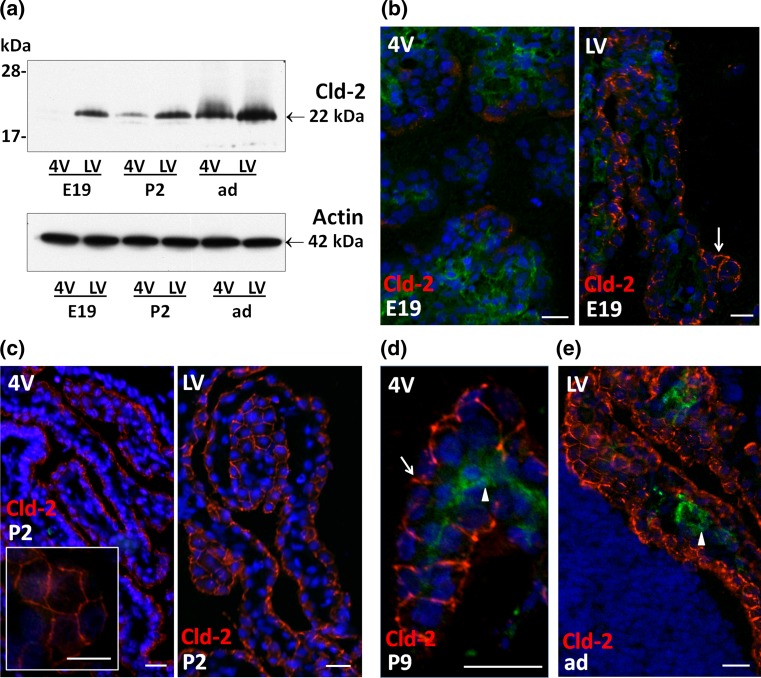
Differential expression of claudin-2 in choroid plexus during rat brain development. **a** Representative Western blot of LVCP and 4VCP dissected from E19, P2, and adult animals (10 μg per lane). Cld-2 expression increased in CPs during development, and was distinctively higher in LVCP than in 4VCP of E19 and P2 animals (*upper panel* band at 22 kDa). Actin used as a loading indicator is shown in the *lower panel* (42 kDa band). **b**–**e** Cld-2 immunoreactivity (*red*) in the developing and adult brain. LVCP and 4VCP sections have been labeled simultaneously and pictures generated with identical camera settings. Cld-2 staining was associated with the epithelium throughout the LVCP of E19 animals (*arrow*), while the staining in 4VCP was faint and patchy, with large portions of the epithelium remaining unlabeled (**b**). In P2 animals (**c**), the staining was more homogeneous in 4VCP and became continuous between epithelial cells (*insert*), as in the LVCP (*right panel*). The epithelial staining was strong in all CPs at P9 (**d** *arrow*) and in adult animals (**e**). In **b**, **d**, **e** double immunolabeling with anti-RECA-1 Ab allowed visualizing the choroidal vessels unlabeled by the anti-Cld-2 Ab (*arrowheads*). DAPI was used for nuclei staining. *Scale bars* 20 μm. Abbreviations as in Fig. 2

Cld-2 expression largely increased throughout development in the 4VCP. A developmental Cld-2 upregulation also occurred in the LVCP to a lesser extent, as mRNA levels in perinatal stages were already higher in this tissue than in 4VCP (Fig. 1a). These differences between either CPs or stages were reflected at the protein level. Western blot analysis showed a signal in LVCP of E19 rats, which became progressively more intense in older animals. Cld-2 was barely detected in 4VCP from E19 rats, and the band increased in intensity in older animals (Fig. 4a). Cld-2 immunoreactivity was associated with all intercellular epithelial junctions in LVCP of E19 rats, but displayed a patchy, discontinuous pattern of lower intensity in 4VCP of the same animals (Fig. 4b). The difference between LVCP and 4VCP was less prominent at P2, as Cld-2 labeling became continuous throughout the TJ network in the latter tissue (Fig. 4c). Cld-2 immunoreactivity was intense and regular in all CPs of P9 and adult rats (Fig. 4d, e). No staining of the MV was observed in the choroidal stroma or the brain parenchyma at any developmental stage.

A mirror image was observed when Cld-11 expression levels were compared between LVCP and 4VCP (Fig. 1b). Cld-11 gene was expressed at a much higher levels in 4VCP than in LVCP at all E19, P2 and P9 stages. In adult animals, Cld-11 expression level decreased in CPs, while it increased largely in the cortex. This likely reflects axonal myelination, Cld-11 being a crucial component of the interlamellar strands in oligodendrocyte myelin sheaths (Morita et al. 1999a). These differences of choroidal expression could not be assessed at the protein level, as a Western blot signal could only be obtained on medulla samples from myelinated brains. No other Cld showed such a difference in expression between LVCP and 4VCP.

### Claudin-9 and -19 are selectively enriched in choroid plexus epithelial cells

**Fig. 5:**
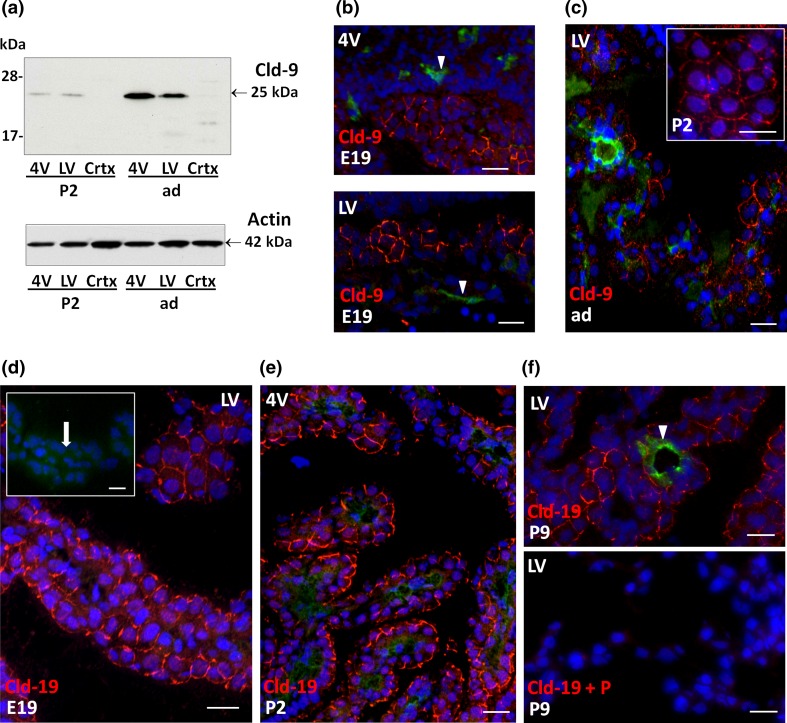
Detection of claudin-9 and claudin-19 in choroid plexus during rat brain development. **a** Representative Western blot of LVCP, 4VCP and cortex dissected from P2, and adult animals (10 μg per lane). Cld-9 protein was enriched in both 4V and LV compared to the cortex, and was higher in adult as compared to P2 animals (*upper panel* band at 25 kDa). Actin used as a loading indicator is shown in the *lower panel* (42 kDa band). **b**, **c** Cld-9 immunoreactivity (*red*) in the developing and adult brain. A distinct signal was associated with the CP epithelial cell membrane in selected areas of both LVCP and 4VCP in E19 animals (**b**). The staining was present at all intercellular junctions in P2 animals (*insert* **c**), and was observed throughout the CPs in adult animals (**c**). It was not associated with parenchymal vessels (*arrowheads*). **d**–**f** Cld-19 immunoreactivity (*red*) in the developing and adult brain. Choroid plexus epithelial cell membranes were distinctly labeled in E19 animals (**d**), displaying a typical honeycomb pattern. The *insert* shows a negative control obtained by omitting the primary antibody. The *arrow* localizes the CP. A strong signal was also observed in the epithelium at later stages, as illustrated for 4VCP in P2 (**e**) and LVCP in P9 (**f** *upper panel*) animals. No choroidal staining was observed when the Ab was preincubated with excess of immunogen peptide (*P*) (**f** *lower panel*). The signal was absent from the choroidal vessels (*arrowhead* **f**). In **b**, **c**, **f**, double immunolabeling with anti-RECA-1 Ab allowed visualizing the choroidal vessels. DAPI was used to stain nuclei. *Scale bars* 20 μm. *Crtx* cerebral cortex. Other abbreviations as in Fig. 2

Among the four additional Clds whose mRNAs were selectively enriched in CPs in comparison to the cortex or MV, Cld-6 displayed higher choroidal expression levels at perinatal than at P9 and adult stages, while Cld-22 was expressed more prominently in both CPs from P2 onwards (Fig. 1b). The protein level and cellular localization of these two Clds could not be investigated due to the lack of adequate antibodies. Cld-9 expression levels in CPs increased from E19 to adult stage (Fig. 1b). This profile was also reflected at the protein level in both 4VCP and LVCP as illustrated by the large increase in Cld-9 signal by Western blot between P2 and adult animals (Fig. 5a). The immunohistochemical analysis revealed a patchy signal for Cld-9 already at E19, which was associated with the epithelium in both CPs (Fig. 5b). The labeling became continuous at all intercellular epithelial junctions in P2 animals (Fig. 5c, insert), and was also observed throughout the CPs in adult animals (Fig. 5c). It was not found associated with parenchymal vessels at any stage (e.g. arrowheads in Fig. 5b).

Cld-19 gene level of expression was close to the adult level throughout development as assessed by qRT-PCR (Fig. 1b). The choroidal localization of the protein determined by IHC showed its association with the epithelial junctions in both LVCP and 4VCP at all stages investigated (Fig. 5d–f). Ab neutralization with the immunogen peptide completely abolished the signal (Fig. 5f). Cld-19 immunoreactivity was absent from the choroidal vessels (e.g. arrowheads in Fig. 5f). It was neither detected in the parenchymal vessels nor in the neuropil at any stage of development (data not shown).

### Claudin-5, -4, and -16 are specific for the blood–brain barrier in the developing brain

**Fig. 6:**
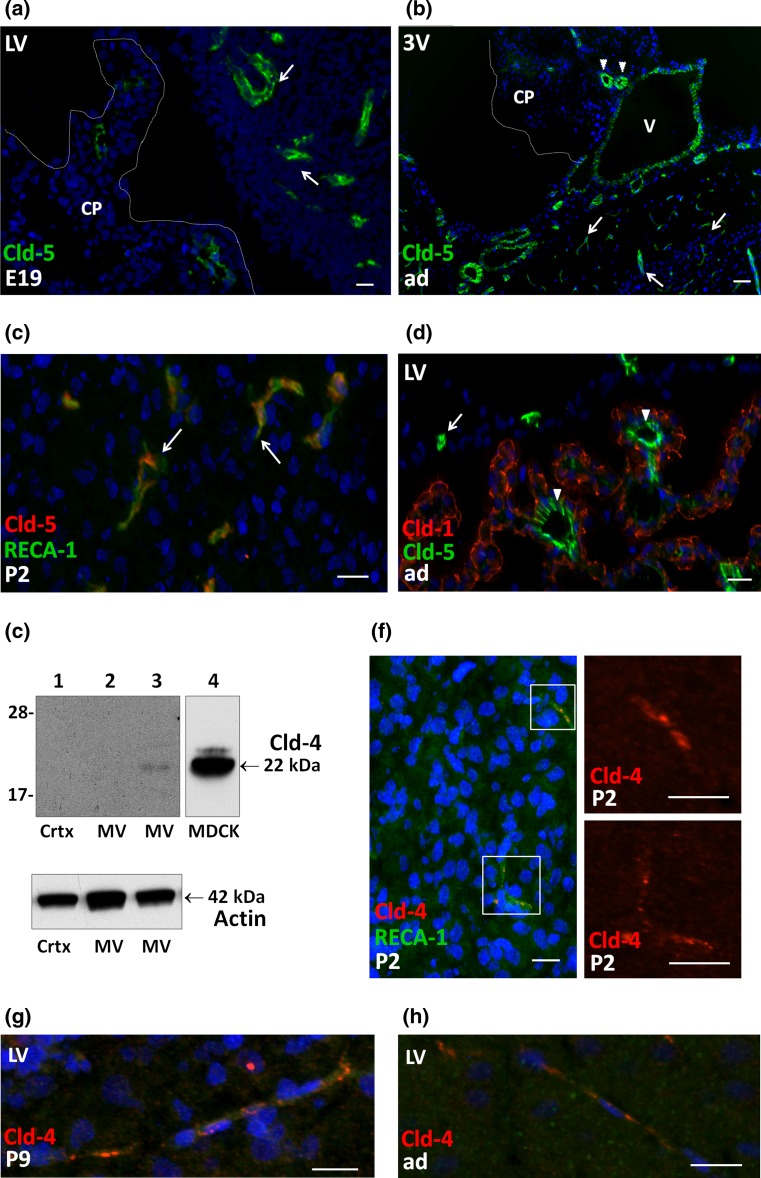
Claudin-5 and claudin-4 are expressed in endothelial cells in the developing brain **a**–**d**. Cld-5 immunoreactivity in the developing and adult brain. Parenchymal vessels are labeled (*green*) in E19 (*arrows* **a**) or adult (*arrows* **b**, **d**) animals. In **c**, the anti-Cld-5 polyclonal antibody was used to show the colocalization of the TJ protein (*red*) with the endothelial marker RECA-1 (*green*) in a P2 animal. In all CPs immunodetection revealed a signal associated with the largest vessels penetrating the choroidal stroma (*arrowheads* **b**, **d**), but not with the choroidal epithelium, labeled in *red* by anti-Cld-1 Ab (**d**). Note that all cisternal vessels such as the vein of Galen are also labeled by anti-Cld-5 Ab. DAPI was used for nuclei staining. **e** Representative Western blot of Cld-4. Brain cortex (*lane* *1* 20 μg) and MV (*lanes* *2* and *3* 40 μg) were isolated from adult animals. *Lane 4* was loaded with MDCK (10 μg) as positive control. A low, but specific Cld-4 signal was detected in MV (*upper panel* *lane 3* band at 22 kDa), but not in cortex or in MV when the primary antibody was omitted (*lane 2*). Actin used as a loading indicator is shown in the *lower panel* (42 kDa band). Due to the high expression of Cld-4 in MDCK cells, *lane 4* was treated separately from the other lanes for HRP detection. **f**–**h** Cld-4 immunoreactivity (*red*) in the developing and adult brain. Double immunolabeling with anti-RECA-1Ab (*green*) allowed visualizing the parenchymal vessels that were positive for Cld-4 in P2 animals (**f** *left panel*). Enlargements of Cld-4 staining associated with the two vessels are shown in the *right panels*. **g**, **h** Parenchymal vessels labeled for Cld-4 in P9 and adult rat brain. DAPI was used for nuclei staining. *Scale bars* 20 μm. *3* *V* choroid plexus of third ventricle, *CP* choroid plexus tissue, *v* vein of Galen. Other abbreviations as in Fig. 2

Cld-5 is a well-known landmark of the BBB. As expected, its expression level was high in cerebral MVs isolated from adult as well as from P9 animals. The more than tenfold difference in Cld-5 gene expression between cortex and MV confirms the endothelial specificity of this protein. In cortical tissue from E19 animals, mRNA expression levels were significantly lower (*p* < 0.001, Tukey’s multiple comparison test) than in other developmental stages (Fig. 1c). As IHC revealed a clear labeling of capillaries by anti-Cld-5 antibodies already at this fetal stage of development (Fig. 6a, b), this most likely results from the lower density of the capillary network in the brain of immature rats (Caley and Maxwell 1970). In contrast, qRT-PCR analysis showed low mRNA levels associated with the choroidal tissue. IHC of Cld-5 within this tissue was performed with the monoclonal antibody and showed that the protein is associated with the larger, penetrating vessels in the choroidal stroma. A fainter signal was also observed in some terminal vessel loops, but no signal was associated with the epithelium in our experimental conditions (Fig. 6c, d).

In addition, our qRT-PCR data identified Cld-16 and Cld-4 as highly enriched in the capillary fractions of both adult and P9 animals compared to cortical or choroidal tissue. To date, no information could be generated concerning Cld-16 protein level or localization in the brain. Cld-4 mRNA levels were very low compared to Cld-5 levels (Fig. 1c). The presence of Cld-4 protein in this fraction was confirmed by Western blot analysis (Fig. 6e) and by IHC in developing and adult animals (Fig. 6f–h).

### Expression of occludin and zonula occludens proteins in barriers of the developing brain

**Fig. 7:**
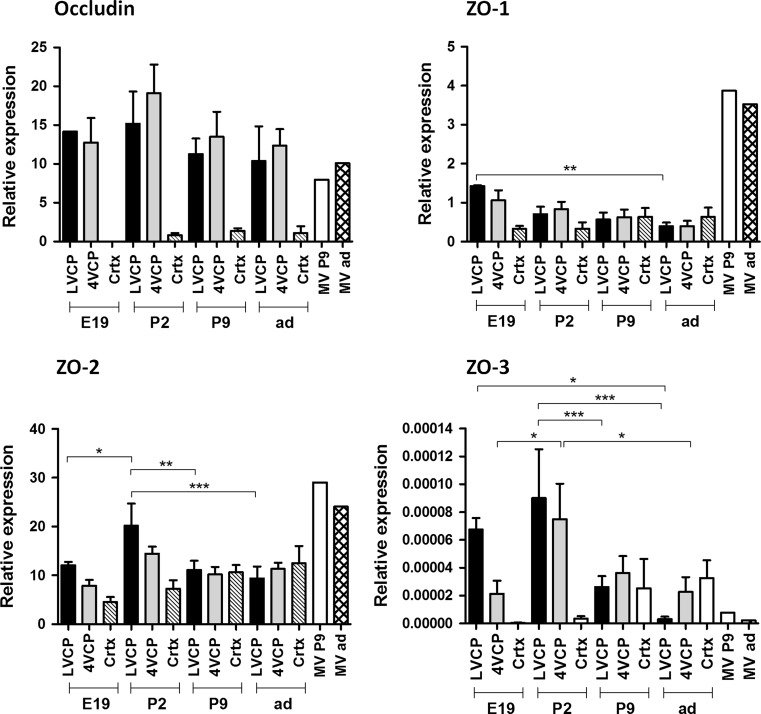
Developmental analysis of occludin and zonula occludens protein transcript levels in choroid plexus, brain cortex and cerebral microvessel of the rat. RT-PCR was performed on tissues sampled at four developmental stages. Moderate (ZO-1, ZO-2) to high (occludin) expression levels were found in both CPs and MV. Very low levels of ZO-3 were detected in both tissues. ZO-3, like occludin were distinctively enriched in blood–brain interfaces; see “Results” for a detailed description of the data. Data normalization, data expression, and statistical analysis are as described in Fig. 1

Occludin is a transmembrane TJ component at both the BBB and BCSFB. Accordingly, qRT-PCR showed high expression levels for this TJ protein in all LVCP, 4VCP and MV preparations compared to cortex. Levels did not change throughout development (Fig. 7). Transcripts for the three ZO proteins that bind Clds through their PDZ domain were found in the CPs throughout development. While the highest expression was seen for ZO-2 with no selectivity among the preparations analyzed, ZO-1 transcript displayed a clear enrichment in the MV preparations, and ZO-3 mRNA, found in both barriers, was selectively enriched in developing CPs, yet at very low levels.

### Claudin-1, -2 and -3 are detected at tight junctions of the blood–CSF barrier in human fetal brain

**Fig. 8:**
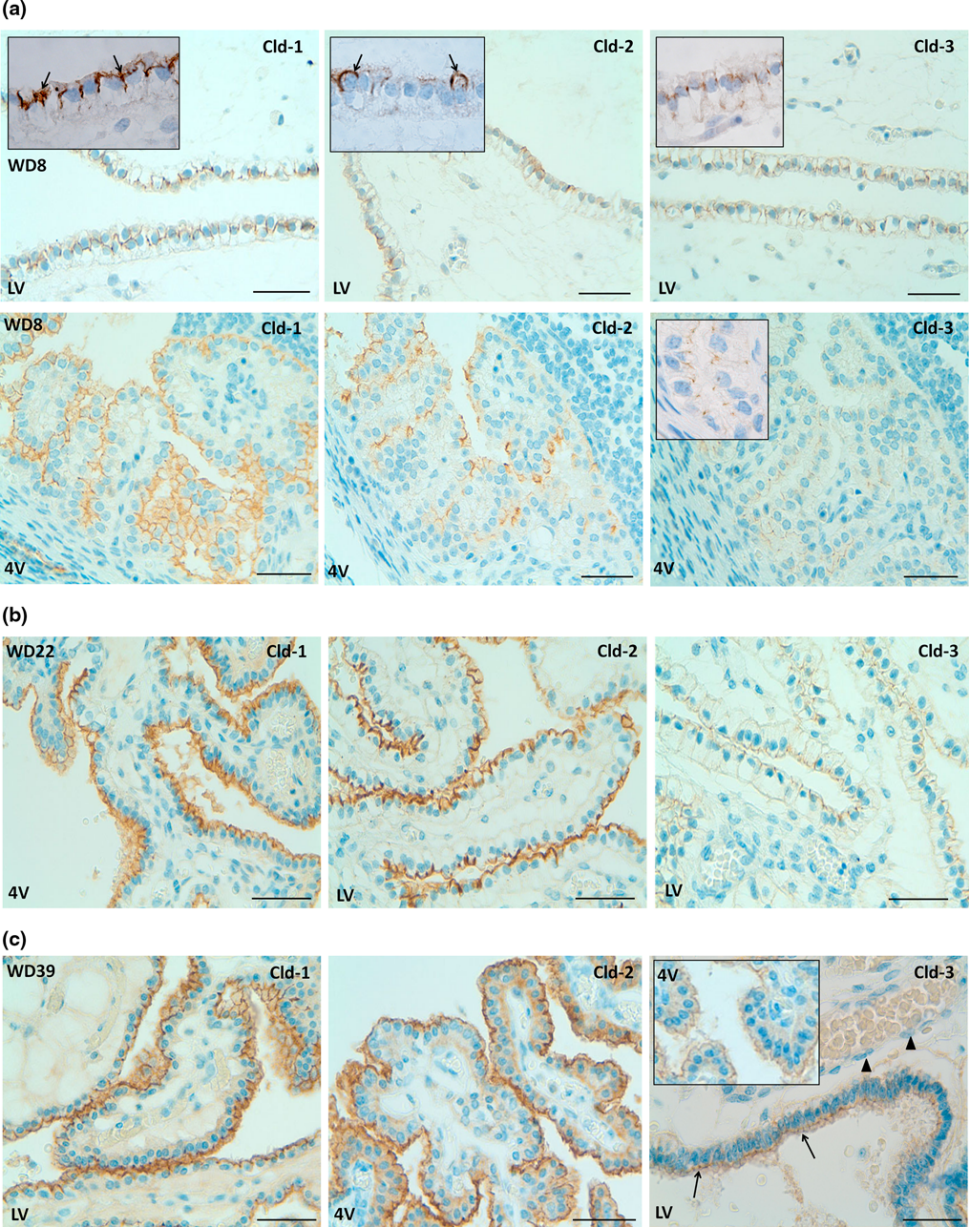
Immunohistochemical detection of claudins in choroid plexuses of developing human brain. Immunoreactivity of Cld-1, -2, and -3 is presented on the *left, central,* and *right panels*, respectively, with hematoxylin counterstaining. **a** Staining of a WD8 fetal brain. Cld-1 labeling, observed in both LVCP and 4VCP, was continuous and localized apically at intercellular junctions of the epithelium (*arrows* in *inserted high magnification micrograph*). Cld-2 immunoreactivity displayed a similar apical, but patchy pattern in both LVCP and 4VCP. The higher magnification (*insert* in LVCP) illustrates the discontinuous labeling (*arrows*). Cld-3 was also detected in the epithelium of both CP (*arrows*). *Inserts* are higher magnifications highlighting the continuous staining pattern. **b** Staining of a WD22 fetal brain. Cld-1 immunoreactivity was present at all epithelial junctions (shown in 4VCP). A continuous apical labeling of the epithelium was observed for Cld-2 (LVCP is shown). Cld-3 labeling was faint but continuous in LVCP epithelial cells. **c** Staining of a WD39 fetal brain. A distinct and continuous labeling of the choroidal epithelium was observed for all Cld-1 (shown for LVCP), -2 (shown for 4VCP), and -3. The continuous signal for Cld-3 was only associated with the epithelium (*arrows*) and not with stromal vessels (*arrowheads* in LVCP). The *insert* shows a similar Cld-3 staining for 4VCP. Abbreviations as in Fig. 2. *Scale bar* 50 μm

The choroidal expression of the three major Clds highly expressed in the rodent BCSFB, Cld-1, -2 and -3, was investigated by IHC on sections obtained from 13 brains of human fetuses and neonates. Table 1 summarizes the clinical data, and indicates the duration of fixation prior to paraffin embedding of the tissues. HPS (not shown) or hematoxylin (Fig. 8a) staining of the tissue, showed morphological differences between LVCP and 4VCP, especially at the earlier stages investigated. LVCPs were characterized by a markedly enlarged core with an “edematous” appearance and were lined with columnar to cuboidal cells with a clear cytoplasm. Conversely, 4VCPs were composed of a thinner papillary core lined with columnar to cuboidal cells with an eosinophilic cytoplasm. Despite these histological differences, a clear apico-lateral staining typical of intercellular choroidal epithelial junctions was observed for Cld-1 throughout the surface of the epithelium in both LVCP and 4VCP already at 8 weeks of development (WD8) which was the earliest fetal stage analyzed (Fig. 8a). This staining was consistently seen at all developmental stages (Fig. 8; Table 1).

A weak and discontinuous apico-lateral Cld-2 immunoreactivity was associated with the CP epithelium in brain sections obtained from WD8 and -10 (Fig. 8a, inserts). This irregular staining became continuous at later stages (Fig. 8b, c; Table 1). A typical TJ-associated labeling was also observed in the choroidal epithelium of both LVCP and 4VCP for Cld-3. This staining was faint but continuous throughout the epithelium. Its intensity varied to some extent from case to case. This was independent of the gestational age and could be related to the duration of fixation (Table 1). The choroidal specificity of Cld-1, -2 and -3 immunoreactivity was strengthened by the absence of staining of the contiguous neuroependyma. Choroidal vessels (e.g. Fig. 8c, arrowheads), and brain parenchyma were negative for these Clds in all 13 cases investigated.

## Discussion

Clds are transmembrane proteins that form the basis of TJ strands in both endothelial and epithelial cells. The identity of the main Clds located in TJs of the BCSFB remained so far uncertain as the anti-Cld-1 antibody used in previous immunohistochemical investigations was later shown to cross-react with other Clds (Wolburg et al. 2003). By combining qRT-PCR analyses with the use of specific antibodies that discriminate between Cld-1, -2 and -3, we have been able to demonstrate that besides Cld-2, already identified in CP (Lippoldt et al. 2000), both Cld-1 and Cld-3 participate into the molecular structure of junctions between choroidal epithelial cells in the rat BCSFB. Cld-1 displayed the highest expression among all Clds in the CPs. Cld-1, -2, and -3 were highly enriched in both LVCP and 4VCP, compared to brain capillaries or brain tissue, and located at the choroidal epithelial junctions.

In addition to these three proteins, and to Cld-11 previously described in mouse (Wolburg et al. 2001), this work identifies Cld-6, -9, -10, -12, -19, and -22 as additional Clds expressed in the CP. The expression of Cld-6, -9, -19 and -22 was highest in the choroidal tissue compared to MVs and cortex, especially during perinatal stages of development. In line with their involvement in TJ complexes at the BCSFB, immunoreactivity of Cld-9 and -19 was localized at intercellular junctions of the epithelium in both LVCP and 4VCP. The large number of Clds associated with the choroidal tissue at the stage E19, and the concurrent expression of occludin and of ZO proteins necessary for the recruitment of Clds to the tight junction strands (Umeda et al. 2006), are in line with the apparent ability of the epithelial junctions to impede the diffusion of both large and small organic compounds, such as proteins and low molecular weight dextrans at early stages of development in *Monodelphis domestica* (Ek et al. 2003, 2006) and in rat (Johansson et al. 2006). Taken together, these data support the concept that a barrier to paracellular transfer between blood and CSF is essential early during development (Johansson et al. 2008b; Saunders et al. 1999, 2012).

The sealing property of TJs results from homo- or heterotypic interactions between Clds of adjacent cells, as well as from lateral oligomerization of Clds within each membrane into strands. These proteins contain a short intracellular cytoplasmic amino-terminal sequence followed by a large extracellular loop (EL1), a short intracellular loop, a second extracellular loop (EL2) and a longer C-terminal cytoplasmic tail. EL1 influences charge and ion selectivity (see infra), while EL2 is important for both Cld oligomerization and cell–cell adhesion (Colegio et al. 2002; Coyne et al. 2003; Daugherty et al. 2007). The identity and relative proportion of Clds within a TJ complex, and the identity of the different cytoplasmic and accessory proteins with which they interact, determine the degree of tightness of a cellular barrier (Overgaard et al. 2011). It can be low as in the case of the kidney epithelium (Gunzel and Yu 2009) or high as in the microvascular endothelium of the BBB (Huber et al. 2001; Nitta et al. 2003). Clds have been classified into two subcategories. The pore-forming Clds, which include Cld-2, -10, -16, allow the charge-selective paracellular diffusion of inorganic ions, and decrease transcellular electrical resistance. They may also participate in a size-selective diffusion of non-charged molecules. In contrast, the sealing Clds such as Cld-1, -3 -4 -5, -11 restrict the paracellular permeability and are considered as barrier-tightening Clds (Original references listed in Online resource 3. For review, Van Itallie et al. 2006; Krause et al. 2008; Overgaard et al. 2011; Yu et al. 2009). In CP, the relative proportion of the different Clds forming TJs differs between developing and adult animals, suggesting that the functions supported by these junctions are developmentally regulated. We observed in both CPs an increase of Cld-9, -22, and mostly Cld-2 gene expression, confirmed by Western blot for Cld-9 and -2, between the embryonic stage E19 and the later developmental stages. Conversely, the expression of Cld-6 and Cld-3 was significantly down-regulated in adult rats compared to perinatal animals, concomitantly with a decrease in Cld-3 protein level. Cld-3 is considered a sealing component of the TJ towards mono- and divalent ions as well as non-charged small solutes (Milatz et al. 2010), while Cld-2 was described as a monovalent cation-selective pore-forming junctional protein displaying a high selectivity for Na^+^ and K^+^ (Furuse et al. 2001; Amasheh et al. 2002; Yu 2009). Its overexpression in MDCK cells increases the transfer capacity of tight junctions for small (up to 250 Da) organic compounds (Van Itallie et al. 2008). It was also described as a paracellular water channel (Rosenthal et al. 2010). Thus, the mirror image of Cld-2 and Cld-3 developmental expression profile in the choroidal epithelial monolayer suggests that the BCSFB is actually less permeable to selected ions or small molecular weight compounds in developing brain than in adult.

This CP specific developmental regulation of Clds could be related to the CSF secretory function of this tissue. The choroidal CSF secretion rate increases prominently after birth in mammals (Catala 1997). In adult CSF secretion is driven by the apically located Na^+^–K^+^–ATPase and carbonic anhydrase II, which are both up-regulated during perinatal brain development in rat (Johansson et al. 2008a). CSF secretion results from a complex coordinated transport of various inorganic anions and cations across the choroidal epithelial monolayer, leading to transcellular fluxes of Na^+^, Cl^−^ and HCO_3_^−^ from the blood to the CSF. Our current understanding of CSF secretion implies that only the transfer of K^+^, necessary to provide adequate K^+^ levels in the CSF, occurs via the paracellular route (Damkier et al. 2010). Thus, the pore-forming Cld-2, whose upregulation in CP parallels the increased rate of CSF secretion, may be involved in K^+^ paracellular movement. Water movement, which is driven by ion exchanges across the choroidal epithelium, was thought to occur transcellularly through aquaporin1 channels. Recently this view was challenged by the phenotypic analysis of aquaporin1 knockout animals. This and other osmolarity data point to mechanisms other than aquaporin-related pathways in CSF water secretion, and revives the paracellular pathway as a route for water transfer from blood to CSF (reviewed in Damkier et al. 2010). In this context a role for Cld-2 in paracellular water movement across the BCSFB, as observed in MDCK cells (Rosenthal et al. 2010) is another appealing hypothesis that could explain the developmental regulation of this Cld.

The selective expression of Cld-19 in choroidal epithelial cells is another common feature that this barrier shares with the kidney epithelium along with a number of transport systems. In the peripheral organ, Cld-19 associates with Cld-16 in selected areas of the thick ascending limb of the loop of Henle to form pores for cations including Na^+^ and the divalent cations Ca^2+^ and Mg^2+^ (Angelow et al. 2007; Hou et al. 2008, 2009). In contrast, in TJ-like structures of peripheral myelinating Schwann cells, Cld-19 acts as a barrier to cations, and thus achieves the electrical sealing of myelin sheaths within the internodal region, which is necessary to maintain saltatory conduction (Miyamoto et al. 2005). In the brain, we found that Cld-16 is localized at the BBB-forming MVs rather than at CPs. Choroidal Cld-19 is therefore more likely to act as a barrier towards Na^+^ and divalent cations rather than as a pore-forming Cld. This hypothesis is in accordance with the existence of an unidirectional, energy-dependent blood to CSF Ca^2+^ and Mn^2+^ transport across the CP epithelium (Schmitt et al. 2011), whose efficiency depends on a closed paracellular pathway to these cations. The sealing function of Cld-19 towards selected cations at the choroidal tight junctions yet remains to be experimentally demonstrated, and the capacity of Cld-19 to homo- or heterodimerize with Clds other than Cld-16 needs further investigations.

Like Cld-19 in Schwann cells, Cld-11 fulfills electrical sealing functions in myelinating oligodendrocytes (Morita et al. 1999a). Cl-9 acts as a paracellular permeability barrier towards Na^+^ and K^+^ in the cochlea (Nakano et al. 2009). Cld-9 and -11 may have similar functions in the choroidal epithelium and, in conjunction with Cld-19, may confer upon the BCSFB junctions a sealed phenotype towards non-specific cation leakage. The reason for the reverse developmental regulation of Cld-9 and Cld-11 remains however to be clarified.

Another unexpected finding was the reverse ratios in LVCP to 4VCP expression levels for Cld-2 and -11 during the perinatal period. Very few differences between the various CPs have been documented, partly because LVCPs are preferentially studied. Our results point to such differences, whose functional significance remains to be determined. Evaluation of the respective contribution of LVCPs and 4VCPs to CSF secretion around birth could support the potential involvement of Cld-2 in CSF secretion.

Overall, the developmental differences in tight junction gene expression that we observed at the BCSFB appear to be necessary for physiological adaptation and/or microenvironmental changes during brain development, rather than reflecting an immaturity of the barrier.

The three most highly expressed Clds (Cld-1, -2 and -3) in rat CPs were also examined by immunohistochemistry in CPs of human fetuses of different gestational stages. Human data paralleled the findings observed in rat. Immunoreactivity of all three Clds was localized at the epithelial tight junctions in both LVCP and 4VCP at all stages. Cld-1 and -3 staining was already continuous throughout the intercellular regions of the choroidal epithelium from the earliest WD8 stage. This is in accordance with a previous study showing the apical localization of Cld-1 in CP of human embryos (Anstrom et al. 2007). Cld-2 signal displayed a patchy pattern at that early time before it became continuous around WD16. In human as in other mammals, CPs appear in the order of the fourth, then lateral ventricle. Given that they form around the WD 6–7 stage (Shuangshoti and Netsky 1966), our data imply that a restrictive and selective barrier exists in human fetuses between blood and CSF, very rapidly after the CP primordium starts to differentiate. The research focus on Clds has always been on their role in tight junction structure. However, the plethora of Clds revealed by this study, their differential expression in the two types of CP and their age-related regulation suggest that there remain other so far undiscovered functions for some of these Clds.

With respect to TJ proteins at the BBB, we showed that, in addition to the previously reported Cld-5 (Morita et al. 1999b), brain parenchymal MVs selectively express Cld-16, as well as Cld-4 at lower levels. Both Cld-5 and -4 genes, considered as sealing Clds, were expressed in E19 or P2 animals, in line with the observation that the BBB is already impermeable to polar tracers in the developing brain (Ek et al. 2006). We also detected mRNAs for Cld-3, -9, -10, -12 and -22 in MVs prepared from P9 and adult rat brain, in accordance with the previous molecular analysis realized on mouse brain endothelial cells (Nitta et al. 2003; Ohtsuki et al. 2008). Cld-1 was not detected in brain vessels at any stage of development, neither in rat nor in human. This confirms the findings of a previous study performed on human embryos (Anstrom et al. 2007). The significance of the pore-forming Cl-10 and Cl-16 expression at the BBB, characterized by a high transendothelial electrical resistance is unclear.

Much evidence exists for modulation of Clds in response to inflammation, infection or oxidative stress, gathered from in vitro cell cultures or in vivo studies in the adult. Cld-3 was down-regulated in the inflamed BBB in a mouse model of multiple sclerosis (Wolburg et al. 2003), and was shown to be specifically sensitive to H_2_O_2_ exposure in epithelial gastric cells (Hashimoto et al. 2008). Oxidative stress induced by intracerebral HIV gp120 protein injection in the caudate putamen of rats increased the expression of matrix metalloproteinases-2 and -9 which in turn induced a loss of Cld-5 immunoreactivity (Louboutin et al. 2010). Clds can also play a role in infectious diseases by mediating toxin deleterious effects or triggering virus cellular entry. A receptor site for *Clostridium perfringens* enterotoxin, present on the EL2 segment of Cld-3 and Cld-4, initiates the process leading to ileal epithelial cell dysfunction and necrosis. Cld-1, and possibly Cld-6 and -9 have been identified as co-receptors interacting with the tetraspanin protein CD81 to mediate the internalization of hepatitis C virus in the liver (Evans et al. 2007; Zheng et al. 2007). This mechanism may hold true for the brain or CSF in which the virus has been found (Wilkinson et al. 2009). Brain damage induced by perinatal injury often involves oxidative stress, inflammation or infection episodes (Dammann and Leviton 1999; Hagberg et al. 2012). An alteration of choroidal epithelial tight junctions triggered by such events would lead to an impairment of the BCSFB and thus of the cerebral homeostasis that is necessary to normal brain development. As perinatal injury can have dramatic consequences for brain development, a careful evaluation of Cld deregulation at blood–brain interfaces is therefore called for.

In summary, the expression pattern of Clds, occludin and zonula occludens proteins in LVCP and 4VCP has revealed a high degree of complexity in the control of the permeability at the BCSFB. The developmental expression profiles indicate an early establishment of this barrier, a finding that was confirmed in human fetuses. Variations in Cld choroidal expression between CPs and during development probably reflect changes in selective blood to CSF transport functions during development, and may be crucial for brain protection, especially in the context of perinatal injuries or exposure to toxins and drugs.

## Electronic supplementary material

Below is the link to the electronic supplementary material.

Supplementary material 1 (PDF 68 kb)

Supplementary material 2 (PDF 155 kb)

Supplementary material 3 (PDF 89 kb)
