# Supplementary material for: Complexity and developmental changes in the expression pattern of claudins at the blood–CSF barrier
Source: Histochem Cell Biol. 2012 Aug 11;138(6):861–79. doi: 10.1007/s00418-012-1001-9 (PMC3483103; doi:10.1007/s00418-012-1001-9)
Supplement: Supplementary file 1 — Supplementary material 1 (PDF 68 kb) [file 418_2012_1001_MOESM1_ESM.pdf]

Complexity and developmental changes in the expression pattern of claudins at the blood-CSF barrier

Histochemistry and Cell Biology
I Kratzer, A Vasiljevic, C Rey, M Fevre-Montange, N Saunders, N Strazielle, JF Ghersi-Egea
Inserm U1028, Lyon Neuroscience Research Center, Neurooncology & Neuroinflammation Team, Lyon-1 University, F-69000, France
E-mail: jean-francois.ghersi-egea@inserm.fr

ESM_1 List of primers, product length and $MgCl_2$-concentration used for qRTPCR

**Gene name**	**Forward primer 5‘→ 3‘**	**Reverse primer 5‘→ 3‘**	**size (bp)**	**$MgCl_2$**
AraB (*E. coli*)	ATCCCCTGATCGGTAAAGCA	ACGCCTGAAAGGGGTGATTA	126	4 mM
Claudin 1	GCTAAGCTGCTAACCCTGTGG T	TCT GGC AAG TCT AGC AGT TTG TG	202	3 mM
Claudin 2	GGCTACCAGGCCCAGCCTCT	TGC CTG TAA CCC TGG GGG CA	193	3 mM
Claudin 3	ACTACCAACCGTCGATGTACCC	CCA TTC GAC TTG GAC AGT TCC T	188	3 mM
Claudin 4	TGGACAGGTTTGAGGGAAGG	GGTCCTTCTGAAGAGGGAGGG	193	4 mM
Claudin 5	TACATGCTAACCTGAAAGGGCA	AGG AGG AAG GCA ACC CTT CTA A	196	4 mM
Claudin 6	ACGCCCTCGGACAAAGCTGAC	ACATCGGCAGGGCACAGGAC	135	3 mM
Claudin 9	GCTGTGATGGGCTGAAGGCTTCC	TCA ACA GGG TTG GGA GCA CCG	199	3 mM
Claudin 10	CTTGGAAGGACCATTGTGAG	GTACAATGGACCCACGTCAG	180	3 mM
Claudin 11	TGAAGGAACTGAACCAAGCAGA	AAA CAG CAC TGC TTC AAG ATC G	208	3mM
Claudin 12	TTTAAGTGTTCCAGATTGGGCA	AGG TTG TCT GAC ACA CTG GCT C	181	4mM
Claudin 16	TCGCAGGTACCCCGGGAATCA	CGA GAG AGG AGC GCT CGA CG	72	3mM
Claudin 19	CAAGACCCCGCCCACACGTG	AGC ACC GTG CGC TGA GAT CC	195	3mM
Claudin 22	TGCTTTATTTACTGGTGCCCTG	CTTCCCTCCTGTGGCTATCATC	184	3 mM
Occludin	GACTGGGTCAGGGAATATCCACC	AGC AGC AGC CAT GTA CTC TTC AC	192	3mM
ZO-1	TTCTTGCAAAGTATCCCTTCTG	CCA CAA AAG AAA TCC TTT CAC A	165	4mM
ZO-2	CTGAATGCATGAGGATCTTGGA	CCA CAA AGT CAG AGG CTT GAG A	188	4mM
ZO-3	GGGAACAGCACACGGCCACAC	CGG CCC TCC AGG TAC CAC GTC	122	4mM
