# Supplementary material for: Complexity and developmental changes in the expression pattern of claudins at the blood–CSF barrier
Source: Histochem Cell Biol. 2012 Aug 11;138(6):861–79. doi: 10.1007/s00418-012-1001-9 (PMC3483103; doi:10.1007/s00418-012-1001-9)
Supplement: Supplementary file 2 — Supplementary material 2 (PDF 155 kb) [file 418_2012_1001_MOESM2_ESM.pdf]

Complexity and developmental changes in the expression pattern of claudins at the blood-CSF barrier

Histochemistry and Cell Biology

I Kratzer, A Vasiljevic, C Rey, M Fevre-Montange, N Saunders, N Strazielle, JF Ghersi-Egea
Inserm U1028, Lyon Neuroscience Research Center, Neurooncology & Neuroinflammation Team, Lyon-1 University, F-69000, France.
E-mail: jean-francois.ghersi-egea@inserm.fr

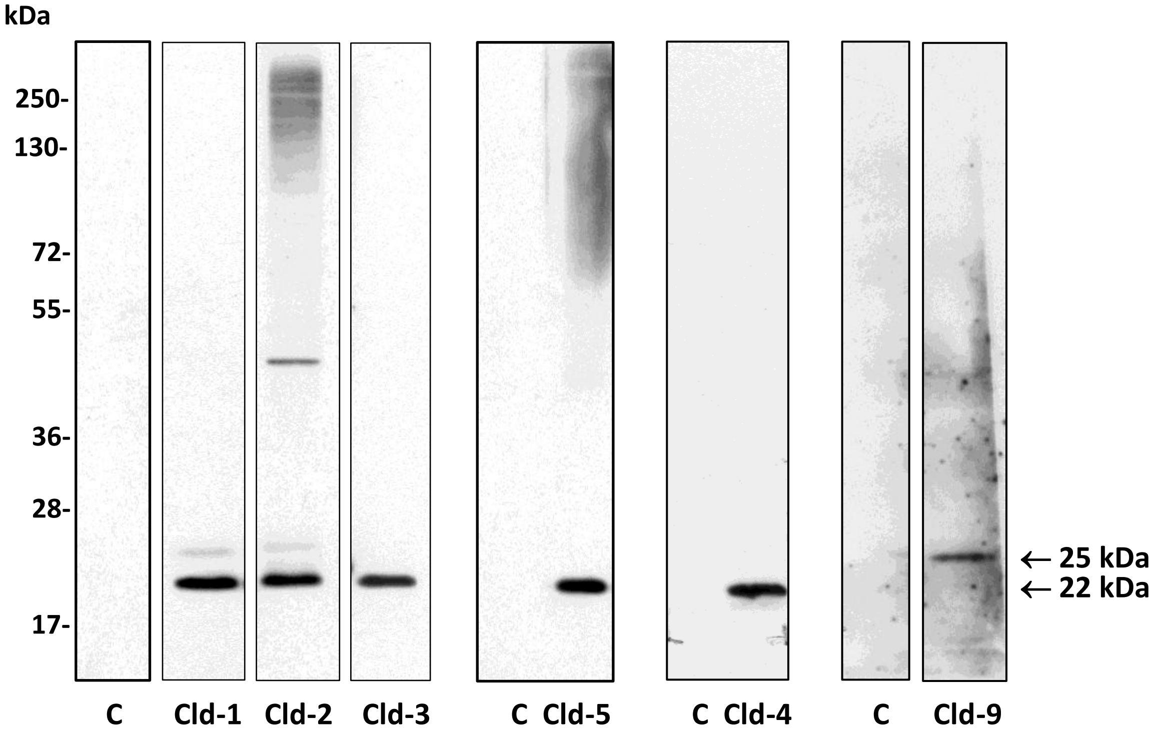

ESM_2 Western blot analysis of Antibody specificity

Full length electrophoretic profile are shown for homogenates of lateral ventricle choroid plexuses (10 µg) used to test the polyclonal rabbit anti-Cld-1, Cld-2, Cld-3, and Cld-9 Ab, of brain microvessel preparations (5 µg) used to test the monoclonal anti-Cld-5 Ab, and of MDCK (10 µg) to test the polyclonal anti-Cld-4 Ab. The specificity of the Ab was confirmed by the detection of a strong band around 25 kDa for Cld-9 and 22 kDa for the other Clds. In addition, Cld-2 Ab revealed a second band at 45 kDa, which may result from dimer formation. C: control obtained in identical ECL conditions by omitting the primary antibody
