# Supplementary material for: Complexity and developmental changes in the expression pattern of claudins at the blood–CSF barrier
Source: Histochem Cell Biol. 2012 Aug 11;138(6):861–79. doi: 10.1007/s00418-012-1001-9 (PMC3483103; doi:10.1007/s00418-012-1001-9)
Supplement: Supplementary file 3 — Supplementary material 3 (PDF 89 kb) [file 418_2012_1001_MOESM3_ESM.pdf]

Complexity and developmental changes in the expression pattern of claudins at the blood-CSF barrier

Histochemistry and Cell Biology
I Kratzer, A Vasiljevic, C Rey, M Fevre-Montange, N Saunders, N Strazielle, JF Ghersi-Egea
Inserm U1028, Lyon Neuroscience Research Center, Neurooncology & Neuroinflammation Team, Lyon-1 University, F-69000, France
E-mail: jean-francois.ghersi-egea@inserm.fr

ESM_3 Tentative classification of claudins as pore-forming or barrier-tightening proteins
Based on *in vivo* (KO-animals) or *in-vitro* cell culture models (reviewed partly in Furuse and sukita (2006) Trends Cell Biol 16:181-188; Krause et al. (2008) BBA 778:631-645.

Barrier-tightening	References
Cld-1	Furuse et al. (2002) J Cell Biol 156:1099-1111.
Cld-3	Milatz et al (2010)Biochim Biophys Acta 798:2048-2057
Cld-4	Van Itallie et al. (2001) J Clin Invest 107:1319-1327.
Cld-5	Morita et al. (1999) J Cell Biol 147:185-194.<br>Nitta et al. (2003) J Cell Biol 161:653-660.
Cld-9	Nakano et al. (2009) PLoS Genet 5:e1000610.
Cld-11	Gow et al. (1999) Cell 99:649-659.<br>Morita et al. (1999) J Cell Biol 145:579-588.
Cld-14	Ben-Yosef et al. (2003) Hum Mol Genet 12:2049-2061.
Cld-19	Miyamoto et al. (2005). J Cell Biol 169:527-538.

Pore-forming	Reference
Cld-2	Furuse et al (2001) J Cell Biol 153:263-272.<br>Muto et al (2010) PNAS, 107:8à11-8016.
Cld-6 ?	Turksen and Troy (2002) Development 129:1775-1784.
Cld-10	Van Itallie et al. (2006) Am J Physiol Renal Physiol 291:F1288-1299.
Cld-12	Fujita et al. (2008) Mol Biol Cell 19:1912-1921.
Cld-16	Hou et al. (2007) J Biol Chem 282:17114-17122.
Cld-16 in association with Cld-19 in kidney	Angelow et al. (2007) Am J Physiol Renal Physiol 293:F166-177.<br>Hou et al. (2008) J Clin Invest 118:619-628.

The definite functions of Cld-6 and Cld-22 are currently unknown.
